# Three-dimensional thoracic and pelvic kinematics and arm swing maximum velocity in older adults using inertial sensor system

**DOI:** 10.7717/peerj.9329

**Published:** 2020-07-07

**Authors:** Xin Fang, Zhongli Jiang

**Affiliations:** 1 School of Rehabilitation Science, Nanjing Normal University of Special Education, Nanjing, Jiangsu, China; 2 Department of Rehabilitation Medicine, The First Affiliated Hospital of Nanjing Medical University, Nanjing, Jiangsu, China

**Keywords:** Torso motion, Arm swing, Inertial sensor, Old adults, Regression model

## Abstract

Understanding characteristics of torso motion and arm swing of older adults is important. A comprehensive database of three-dimensional thoracic and pelvic kinematics and arm swing maximum velocity of older adults during overground walking is still lacking. Moreover, the relationships between these variables are not fully understood. Therefore, we investigated age and gender effects of three-dimensional thoracic and pelvic ranges of motion and arm swing maximum velocity in 113 healthy old adults (aged 60–89 years) in a 2-min walk test using APDM Movement Monitoring inertial sensor system by two-way ANOVA, and post hoc Bonferroni correction was applied for multiple comparisons between age groups. A paired *t*-test was used to study the side preference of arm swing maximum velocity. The relationships between variables were investigated via multiple linear regression models. In general, thoracic and pelvic motions showed reduced amplitude with aging. Gait speed, pelvis coronal plane motion and arm swing maximum velocity significantly declined with age. Only the pelvic sagittal plane motion showed a gender main effect. Coronal plane motions of the thorax and pelvis were closely associated, as were sagittal plane motions. Thoracic coronal plane motion was the significant variable influencing pelvic transverse plane motion and vice versa. Gait speed, pelvic coronal and transverse plane motions and thorax sagittal plane motion were significant independent variables that influenced dominant arm maximum velocity. A larger maximum velocity was seen in the left arm. This investigation is valuable for better understanding of gait phenomena and will contribute to identification of gait dysfunction and development of rehabilitation measures.

## Introduction

There is a growing interest among researchers in torso movements and the arm swing during gait. Despite having a small range of motion, the torso plays an important role in human locomotion. Torso segments are known to move in three dimensions (i.e., sagittal, coronal and transverse planes) during normal walking, achieving gait efficiency and overall balance. During gait, the maximal range of motion in the frontal plane of the trunk is reached at toe off. The trunk moves from flexion to extension during the double support period and from extension to flexion during the single support period (Cromwell et al., 2001), counterbalancing the leg swing in the sagittal plane (Chung et al., 2010). In the transverse plane, as gait speed increases, the pelvis rotates earlier to shorten the in-phase duration between the thorax and pelvis towards the counter-rotation (Yang et al., 2013), decreasing the rotational momentum to smoothen the gait (Stokes, Andersson & Forssberg, 1989). The arm swing is believed to be an integral part of the bipedal gait, is generated mostly in a passive pattern, and is stabilized by active muscle control (Meyns, Bruijn & Duysens, 2013). The arm swings opposite to leg motion, which serves to reduce body angular momentum, decrease the vertical ground reaction moment and metabolic energy, improve gait efficiency and optimize dynamic stability (Meyns, Bruijn & Duysens, 2013; Collins, Adamczyk & Kuo, 2009).

Inertial sensor-based gait analysis systems are portable and easy to use not only applicable in the laboratory and clinical settings, but also in daily living environments including community and family settings (El-Gohary et al., 2013; Mancini et al., 2015) compared with traditional laboratory-based equipment (e.g., optical 3D motion capture system). Nowadays, inertial sensor-based gait analysis systems are more and more widely used to efficiently capture and analyze quantitative gait data. Prior investigations of thoracic and pelvic ranges of motion during walking were based on small sample sizes (Chung et al., 2010; Stokes, Andersson & Forssberg, 1989; Leardini et al., 2013; Macpherson et al., 2016; Mirelman et al., 2015; Shishov et al., 2017; Staszkiewicz et al., 2012; Van Emmerik et al., 2005; Whittle & Levine, 1999), of which studies involving old subjects were relatively scarce (Mirelman et al., 2015; Shishov et al., 2017; Van Emmerik et al., 2005). Besides, only one of these studies was conducted with inertial sensors (Mirelman et al., 2015). A comprehensive database of three-dimensional ranges of motion of the thorax and pelvis of older adults during overground walking has not been reported.

While arm swing amplitude, asymmetry and variability have been well documented (Mirelman et al., 2015, 2016; Huang et al., 2012; Killeen et al., 2017a, 2017b, 2018; Lewek et al., 2010; Ospina et al., 2018; Plate et al., 2015; Roggendorf et al., 2012; Sterling et al., 2015; Zampieri et al., 2010; Ford, Wagenaar & Newell, 2007), only a few studies were related to arm swing velocity (Ospina et al., 2018; Sterling et al., 2015; Zampieri et al., 2010; Salarian et al., 2010). Using arm swing maximum velocity (degrees/second) to gauge range of motion can provide a quantitative measurement for capturing important dynamical features of gait function. For instance, the peak arm swing velocity on the more affected side (MAS) was significantly slower in early-to moderate, untreated PD during instrumented Timed Up and Go (iTUG) test. The receiver operating characteristics (ROC) analysis revealed an area under the curve (AUC) of 0.958 for peak arm swing velocity of MAS, which was a higher discriminative value than arm swing range of motion of MAS of 0.910 (Zampieri et al., 2010). Early PD showed significant reductions in arm swing speed compared with control subjects (Ospina et al., 2018; Salarian et al., 2010). The angular velocity amplitude of the slower-swinging arm of early-stage PD was reduced in the OFF-medication state and increased after dopaminergic treatment compared to controls (Sterling et al., 2015). However, to date, a comprehensive database of the arm swing maximum velocity (i.e., peak angular velocity of the arm in the sagittal plane) of older adults during overground walking is still lacking.

Age and gender effects are important influencing factors to be considered. Generally, the values of the torso angular ranges of motion during walking are small and not change obviously as age advances in young period (Macpherson et al., 2016; Staszkiewicz et al., 2012; Whittle & Levine, 1999). However, as people grow older, their body flexibility and walking ability decline, which may give rise to evident changes in torso motions. These changes in torso movement may be associated with risk of falls and the inability to maintain balance in older age (Mirelman et al., 2015). For example, older adult fallers displayed reduced total range of motion in the transverse and frontal planes of the pelvic and thoracic regions compared to non-fallers (Shishov et al., 2017). As to gender impact, no gender differences were found in the three-dimensional kinematics of the thorax and pelvis in young participants (Leardini et al., 2013). With respect to arm swing, the arm swing amplitude decreased with age, and their relationship was mediated by gait speed (Mirelman et al., 2015). Multivariate regression model showed gait speed, arm swing amplitude of the dominant arm, arm swing asymmetry and axial rotation jerk were independently associated with aging (Mirelman et al., 2015). Concerning gender and side preference of arm swing, a study on the treadmill indicated a tendency of larger arm swing amplitudes in women and in the left side (Plate et al., 2015). Besides, the mean index of asymmetry of arm swing showed a trend towards left side preference in 16 healthy young subjects (Kuhtz-Buschbeck et al., 2008). However, age and gender effects of three-dimensional thoracic and pelvic ranges of motion and arm swing maximum velocity as well as the side preference of arm swing maximum velocity in healthy old adults are largely unknown. The present study addressed these issues.

In addition, although previous study using multivariate regression model revealed close correlations between some torso and arm swing variables (Mirelman et al., 2015), the relationships of three-dimensional thoracic and pelvic motions and the arm swing maximum velocity have not been fully understood. This study was designed to address this gap. The relationships between these variables were also explored.

## Methods

### Subjects

A total of 113 healthy old subjects (aged 60–89 years) from local communities and elderly centers were included in the study. Eligible participants were in good health and were able to walk independently without aid. Health conditions that would interfere with the gait pattern and arm swing were excluded, including the following: neurological and musculoskeletal pathologies, orthopedic disorders, cardiopulmonary diseases, shoulder limitation and a history of spinal or pelvic surgery. All participants were right-handed based on the Edinburgh Handedness Inventory (Veale, 2014). The measurement protocol adhered to the Helsinki declaration. The study was approved by the ethical committee of the first affiliated hospital of Nanjing Medical University (Approved No. 2017-SR-002), and all of the participants provided written consent prior to participation.

### Procedures

Anthropometric information (height and weight) was collected via a health scale (xiheng®, RGZ-120-RT). The subjects were required to wear comfortable clothes and they were free to swing their arms. Walking shoes (not high-heeled shoes or slippers) were worn. Subjects were required to walk back and forth on a 7-m straight walkway at their natural comfortable speed for 2 min. Six inertial sensors (Opal APDM, Portland, Oregon) were fixed on the following body sites via elastic straps (Fig. S1): sternum (on top of the sternum, with the top of the sensor located at the point separating the body of the sternum and the manubrium in reference to the thorax); center of the lower back (lumbar vertebrae 5 in reference to the pelvis) (Buckley et al., 2017); each wrist; and each foot. Inertial sensors were recalibrated and synchronized before use. A 30-s warm up (walking back and forth on the walkway at natural comfortable speed) was performed to help the participants become familiar with the test. Data acquisition was conducted by an experienced operator.

### Apparatus

Gait parameters were measured with APDM Movement Monitoring inertial sensor system. Body-worn inertial sensors (Opals) incorporate tri-axial accelerometer, tri-axial gyroscope, and magnetometer. Signal processing and calculation of gait parameters were performed via the integrated Mobility Lab™ software that contains algorithms validated by gold-standard methods and can reliably quantify information of balance and gait (El-Gohary et al., 2013; Salarian et al., 2010; Morris et al., 2019; Trojaniello et al., 2015; Lanovaz et al., 2017; Mancini et al., 2011). Data were recorded at a sampling rate of 128 Hz. Turns were detected with gyroscopes in the thorax and pelvis sensors with a mathematical model and filtered out of data analysis. In addition, during steady-state walking, data from gait cycles in which subjects decelerated into or accelerated out of turning cycles were also filtered by identifying and omitting data that departed by three or more standard deviations from the mean (Hollman et al., 2016). The walking condition was set to 2 min which included adequate gait cycles (about 108 gait cycles for each participant) to produce stable mean value of each studied parameter. Gait speed is defined as the forward distance traveled during the gait cycle (stride length), divided by the gait cycle duration (stride time), that is, gait speed(m/s) = stride length(m)/stride time(s). Three-dimensional thoracic and pelvic ranges of motion (i.e., coronal, sagittal and transverse planes) were collected through gait cycles based on an integration of the gyroscope (after correction for DC offset). The maximum velocity in the arm swing was provided by the tri-axial gyroscope and calculated for the dominant and non-dominant arms. More information about the calibration and orientation estimation of Opal sensors, joint angle estimation, and angular velocity calculation are available on the website: https://support.apdm.com/hc/en-us.

### Statistical analysis

Subjects were divided into three age groups by decade (Senden et al., 2009). A descriptive analysis was conducted for all of the variables. A normal distribution of each variable was assessed with the Kolmogorov–Smirnov test. The impacts of age and gender were evaluated by a two-way ANOVA, and post hoc Bonferroni correction was applied for multiple comparisons between age groups. To compare the difference between left and right arm swing maximum velocity, a paired *t*-test was used. A stepwise multiple linear regression model was employed to identify each dependent measure with significant independent measures to assess the relationships between the variables under study (gait speed, thoracic and pelvic motions in coronal, sagittal and transverse planes, and arm swing maximum velocity of the dominant arm). Multicollinearity between independent measures was tested using the variance inflation factor (VIF). Beta values, 95% confidence intervals, *P* values, and standard coefficients, together with the adjusted squared *R*, were presented from each model. All of the statistical tests were performed using SPSS 22.0 software (IBM Corp, Armonk, NY, USA) and were evaluated at the 0.05 level of significance.

## Results

### The demographic and anthropometric information was presented in Table 1

The study sample was composed of 113 old subjects (50 men, 44% and 63 women, 56%) whose ages ranged between 60 and 89 years. The heights were comparable between the three age groups. The weight in the 80–89-year-old age group was significantly lower than that in the 60–69-year-old age group (*P* < 0.001). Men had significantly greater heights and weights than women (*P* < 0.001). No difference was found between the age of men and women in each age group.

**Table 1:** Descriptive characteristics of participants (*n* = 113) grouped by age and gender.

Age groups (years)	Men/Women	Age (years)	Height (cm)	Weight (kg)
60–69	M (*n* = 20)	63.95 (3.47)	171.35 (4.93)	73.48 (9.71)
	W (*n* = 25)	66.88 (2.56)	158.48 (5.09)	63.66 (11.05)
	Total (*n* = 45)	65.58 (3.31)	164.20 (8.15)	68.02 (11.48)
70–79	M (*n* = 16)	75.44 (2.56)	170.25 (6.27)	67.94 (9.13)
	W (*n* = 18)	74.33 (2.20)	156.89 (6.20)	61.00 (8.94)
	Total (*n* = 34)	74.85 (2.40)	163.18 (9.14)	64.26 (9.56)
80–89	M (*n* = 14)	83.29 (2.23)	170.29 (7.78)	64.86 (10.50)
	W (*n* = 20)	84.75 (2.63)	155.20 (5.03)	54.53 (8.64)
	Total (*n* = 34)	84.15 (2.55)	161.41 (9.76)	58.78 (10.63)

**Note:**

All variables are presented as the mean (standard deviation).

### Age, gender and interaction effects on each variable by two-way ANOVA and post hoc comparisons between the age groups (Table 2)

The gait speed, thoracic and pelvic motions, and arm swing maximum velocity by age groups and gender were presented. The effects of age and gender on each variable were investigated with two-way ANOVA. Age main effects were found in gait speed (*F*_2_ = 68.903, *P* < 0.001), pelvic coronal range of motion (*F*_2_ = 7.007, *P* = 0.001), and left (*F*_2_ = 9.855, *P* < 0.001) and right (*F*_2_ = 7.953, *P* = 0.001) arm swing maximum velocity. Only the pelvic sagittal range of motion showed a gender main effect (*F*_1_ = 4.672, *P* = 0.03). No age × gender interaction effect was found in the variables.

**Table 2:** Dataset of the studied variables by age and gender shown as the mean (standard deviation).

	Men/Women	Age groups by years			Age effect	Gender effect	Interaction effect
		60–69 (A)	70–79 (B)	80–89 (C)			
Gait speed (m/s)	M	1.12 (0.13)	1.01 (0.17)	0.69 (0.21)			
	W	1.17 (0.14)	0.98 (0.16)	0.71 (0.19)			
	Total	1.15 (0.13)	0.99 (0.17)	0.70 (0.20)	<0.001	0.65	0.56
	Mean difference (95% CI)	A–B***	A–C***	B–C***			
		0.15 [0.06–0.25]	0.44 [0.35–0.54]	0.29 [0.19–0.39]			
Pelvis/coronal range of motion (°)	M	5.41 (1.67)	4.57 (1.24)	3.93 (1.33)			
	W	5.39 (2.22)	5.55 (1.75)	4.00 (1.49)			
	Total	5.40 (1.97)	5.09 (1.59)	3.97 (1.41)	0.001	0.30	0.39
	Mean difference (95% CI)	A–B	A–C**	B–C*			
		0.31 [−0.63 to 1.25]	1.43 [0.49–2.37]	1.12 [0.11–2.13]			
Pelvis/sagittal range of motion (°)	M	3.94 (0.89)	3.67 (1.00)	3.84 (0.96)			
	W	4.56 (0.95)	4.16 (0.75)	3.81 (0.70)			
	Total	4.28 (0.97)	3.93 (0.90)	3.82 (0.80)	0.08	0.03	0.26
	Mean difference (95% CI)	A–B	A–C	B–C			
		0.35 [−0.13 to 0.84]	0.46 [−0.03 to 0.95]	0.11 [−0.41 to 0.62]			
Pelvis/transverse range of motion (°)	M	8.70 (2.35)	7.16 (2.02)	7.33 (1.98)			
	W	8.00 (2.96)	7.75 (2.29)	7.98 (2.32)			
	Total	8.31 (2.70)	7.47 (2.16)	7.71 (2.18)	0.23	0.69	0.37
	Mean difference (95% CI)	A–B	A–C	B–C			
		0.84 [−0.49 to 2.16]	0.60 [−0.73 to 1.93]	−0.24 [−1.66 to 1.18]			
Thorax/coronal range of motion (°)	M	6.06 (1.91)	5.11 (1.64)	5.02 (1.62)			
	W	5.53 (2.03)	5.59 (1.61)	4.82 (1.63)			
	Total	5.77 (1.97)	5.36 (1.62)	4.90 (1.60)	0.10	0.81	0.46
	Mean difference (95% CI)	A–B	A–C	B–C			
		0.41 [–0.57 to 1.39]	0.87 [–0.11 to 1.85]	0.46 [−0.59 to 1.51]			
Thorax/sagittal range of motion (°)	M	4.14 (0.92)	4.20 (1.28)	4.22 (1.04)			
	W	4.80 (0.97)	4.52 (1.04)	4.16 (0.82)			
	Total	4.51 (0.99)	4.37 (1.15)	4.19 (0.90)	0.49	0.11	0.31
	Mean difference (95% CI)	A–B	A–C	B–C			
		0.14 [−0.42 to 0.69]	0.32 [−0.24 to 0.87]	0.18 [−0.41 to 0.78]			
Thorax/transverse range of motion (°)	M	9.75 (2.10)	9.00 (2.25)	10.46 (2.56)			
	W	9.33 (2.28)	10.57 (2.69)	9.58 (2.39)			
	Total	9.52 (2.19)	9.83 (2.58)	9.94 (2.46)	0.68	0.84	0.08
	Mean difference (95% CI)	A–B	A–C	B–C			
		−0.32 [−1.63 to 1.00]	−0.43 [−1.74 to 0.88]	−0.11 [−1.51 to 1.29]			
Left arm/maximum velocity (°/s)	M	176.24 (55.59)	173.99 (76.83)	131.72 (52.82)			
	W	222.05 (85.24)	201.86 (81.73)	130.94 (46.29)			
	Total	201.69 (76.34)	188.74 (79.52)	131.26 (48.30)	<0.001	0.07	0.35
	Mean difference (95% CI)	A–B	A–C***	B–C**			
		12.95 [−25.29 to 51.18]	70.43 [32.19–108.66]	57.48 [16.67–98.29]			
Right arm/maximum velocity (°/s)	M	176.93 (73.79)	162.69 (62.38)	132.55 (63.12)			
	W	211.76 (96.03)	185.98 (88.99)	119.42 (44.89)			
	Total	196.28 (87.68)	175.02 (77.38)	124.83 (52.66)	0.001	0.30	0.37
	Mean difference (95% CI)	A–B	A–C***	B–C*			
		21.26 [−20.39 to 62.92]	71.45 [29.80–113.11]	50.19 [5.73–94.65]			

**Notes:**

* *p* < 0.05.

** *p* < 0.01.

*** *p* < 0.001.

Statistical significance between age groups. 60–69 age group (A); 70–79 age group (B); 80–89 age group (C); Mean difference (95% CI): the mean difference between age groups ((A–B) the mean difference between 60–69 age group and 70–79 age group; (A–C) the mean difference between 60–69 age group and 80–89 age group; (B–C) the mean difference between 70–79 age group and 80–89 age group).

In general, thoracic and pelvic motions showed reduced amplitude with aging. The pelvic coronal range of motion significantly decreased with age (60–69 vs. 80–89, *P* < 0.01; 70–79 vs. 80–89, *P* < 0.05). The thoracic transverse motion showed a slightly upward trend but was not significant. Other variables declined with age but did not reach statistical significance.

The arm swing maximum velocity of the right side significantly declined with age (60–69 vs. 80–89, *P* < 0.001; 70–79 vs. 80–89, *P* < 0.05). A similar result was found on the left side (60–69 vs. 80–89, *P* < 0.001; 70–79 vs. 80–89, *P* < 0.01). The arm swing maximum velocity did not differ significantly between men and women although there was a tendency for women to have larger values than men in 60–69 and 70–79 age groups. However, a paired *t*-test revealed significantly more maximum angular velocity in the left arm than in the right arm (176.60 ± 75.90°/s vs. 168.38 ± 80.64°/s, *P* = 0.04). Further, the individual variability was high.

### Multiple linear regression model to determine each dependent measure with its significant independent measures (Table 3)

All of the variance inflationary factors (VIF) were less than 2 (average VIF 1.18) indicating that there was no multicollinearity among the significant independent variables (EstebanWalker, 2008). The maximum velocity of the dominant arm, the thoracic coronal range of motion and the gait speed were shown to be significant independent variables that affected the pelvic coronal range of motion (41% variance explained). For the pelvic sagittal range of motion, the strongest single predictor retained by the stepwise procedure was the thoracic sagittal range of motion (*R*^2^ = 0.44). For the pelvic transverse range of motion, the thoracic coronal range of motion was a significant independent variable that accounted for 23% of the variance, while for the thoracic transverse range of motion, the thoracic sagittal range of motion was the only variable of significant influence (*R*^2^ = 0.07). The thoracic coronal range of motion increased as the pelvic coronal motion and transverse motion increased, accounting for 37% of the variability. The thoracic sagittal range of motion was found to increase with increasing pelvic sagittal range of motion and the maximum velocity of the dominant arm (half of the variance explained). A regression model showed that gait speed, pelvic coronal and transverse ranges of motion, and thoracic sagittal range of motion were independent predictors accounting for 49% of the variance in the maximum velocity of the dominant arm. All of the models were significant (thoracic transverse range of motion at *P* < 0.01 and the others at *P* < 0.001).

**Table 3:** Multiple linear regression models of gait speed, three-dimensional thoracic and pelvic ranges of motion, and dominant arm maximum velocity.

Dependent measure	Independent measures	Beta values (95% CI)	*P*-value	Standardized coefficients	Adjusted *R*^2^ (overall significance)
Pelvis/coronal range of motion	Dominant arm/maximum velocity	0.006 [0.002–0.010]	<0.01	.284	0.41 (<0.001)
	Thorax/coronal range of motion	0.343 [0.184–0.503]	<0.001	.341	
	Gait Speed	1.463 [0.190–2.736]	0.03	.201	
Pelvis/sagittal range of motion	Thorax/sagittal range of motion	0.598 [0.471–0.724]	<0.001	.665	0.44 (<0.001)
Pelvis/transverse range of motion	Thorax/coronal range of motion	0.658 [0.437–0.878]	<0.001	.490	0.23 (<0.001)
Thorax/coronal range of motion	Pelvis/coronal range of motion	0.399 [0.243–0.554]	<0.001	.401	0.37 (<0.001)
	Pelvis/transverse range of motion	0.268 [0.152–0.385]	<0.001	.361	
Thorax/sagittal range of motion	Pelvis/sagittal range of motion	0.670 [0.518–0.823]	<0.001	.603	0.50 (<0.001)
	Dominant arm/maximum velocity	0.003 [0.002–0.005]	<0.001	.256	
Thorax/transverse range of motion	Thorax/sagittal range of motion	0.640 [0.216–1.063]	<0.01	.273	0.07 (<0.01)
Dominant arm/maximum velocity	Gait speed	125.294 [75.681–174.908]	<0.001	.384	0.49 (<0.001)
	Pelvis/coronal range of motion	10.400 [3.146–17.654]	0.01	.232	
	Thorax/sagittal range of motion	19.270 [8.156–30.384]	0.001	.243	
	Pelvis/transverse range of motion	6.430 [1.653–11.206]	0.01	.191	

## Discussion

This study investigated the impacts of age and gender in a comprehensive database of three-dimensional angular ranges of motion in the thorax and pelvis and arm swing maximum velocity as well as the side preference of arm swing maximum velocity in an older adult population during overground walking using the inertial sensor system. The relationships between these variables by the regression analyses were also explored.

Leardini et al. (2013) studied 30 young subjects and reported no gender differences in the three-dimensional kinematics of the thorax and pelvis. Our study in older adults was in general agreement with this finding, except for the larger pelvic sagittal range of motion in women than in men (Mean Difference (Men–Women) = −0.36°, 95% CI [−0.70° to −0.03°], *P* = 0.03). In general, the thoracic and pelvic ranges of motion decreased with advancing age with the exception of the thoracic transverse motion. This finding was in agreement with Van Emmerik et al. (2005), who reported a significantly increased thoracic rotation in the transverse plane at lower speeds in older individuals compared to the young group. Due to the small value of each variable and the post hoc Bonferroni correction, a significant difference between age groups was only detected in the pelvic coronal range of motion. The pelvic coronal and transverse motions are known to reduce the vertical oscillation of the center of mass to create a more energy efficient gait pattern (Saunders, Inman & Eberhart, 1953). A larger pelvic transverse range of motion gave rise to greater stride length with relatively constant cadence, resulting in increased gait velocity (Nottrodt, Charteris & Wall, 1982). Therefore, reduced pelvic motions in older adults may impact gait stability (Saunders, Inman & Eberhart, 1953) and efficiency with increased energy consumption and low endurance. The underlying causes of decreased trunk motions in older adults may be interpreted as follows: First, Bötzel’s group studied 12 healthy men (age 25–40) walking at three velocities (2, 4, and 6 km/h) on a treadmill and reported that pelvic mean angle of rotation around *z* (earth-vertical axis in the transverse plane) was increased from 9° to 14° as walking velocity increased. The decreased pelvic transverse plane movement with advancing age in old adults may result from reduced gait speed (Bötzel et al., 2018). Second, the degeneration of neural and musculoskeletal systems increases with advancing age, giving rise to greater stiffness in trunk rotation (Van Emmerik et al., 2005). Third, walking stability not only indicates a steady gait but also includes the ability to cope with perturbations (Meyns, Bruijn & Duysens, 2013). In contrast to young adults with flexible trunk movements and a good response to external perturbations, older subjects displayed lesser adaptability with rigid trunk rotations (Van Emmerik et al., 2005). Older individuals tended to reduce the degree of freedom of their body segments while walking to keep a dynamic balance and to prevent falls (Shishov et al., 2017). Thus, developing exercises and gait training to promote trunk flexibility and response to perturbation in older adults may improve gait stability and reduce the incidence of falls.

A close association was also found in thoracic and pelvic sagittal ranges of motion. While Chung et al. (2010) reported that the ranges of the trunk coronal and transverse plane motion appeared to be correlated, we found that the thoracic coronal range of motion was the significant variable influencing the pelvic transverse range of motion, and vice versa. This also concurred with another study (Whittle & Levine, 1999), which suggested “coupling” took place between lateral bend and axial rotation in the lumbar spine. The vector of the spinal muscles or axis of lumbar spinal joint might explain the interconnection between the coronal and the transverse plane motion (Chung et al., 2010), but future investigation will be needed for better understanding of this result.

A study of 60 healthy adults (between 40 and 75 years) walking on a treadmill showed a tendency of larger arm swing amplitudes in women and in the left side but could not reach statistically significance (Plate et al., 2015). Also, the mean index of asymmetry indicated a trend towards left arm-swing preference (Kuhtz-Buschbeck et al., 2008). Our study showed a significantly larger arm swing maximum velocity in the left arm. Possible explanations are environmental/cultural factors or an unclear innate laterality bias irrespective of walking condition or handedness. Presumably, both left- and right-handed populations are constantly exposed to a right-handed environment with objects designed to be manipulated with the right hand, which may promote left-dominant arm swing, with the right arm “primed” for action (Killeen et al., 2018). Although the difference between genders was not significant, women in 60–69 and 70–79 age groups also tended to have larger values than men. Older adults attempted to employ lower walking velocity to compensate for the impaired balance. A two-way ANOVA showed that the gait speed and arm swing maximum velocity decreased with age in older adults. The arm swings out of phase with the leg largely in a passive way to reduce the body angular momentum (Bruijn et al., 2008). Moreover, multiple regression analyses revealed that gait speed was the most important factor that influenced the dominant arm maximum velocity during walking. Thus, the relationship between age and arm swing velocity may be mediated by gait speed. In addition, the dominant arm maximum velocity increased with increasing pelvic coronal and transverse motions and the thoracic sagittal range of motion during walking. When humans walk, the pelvis rotates with each step to create body angular momentum around the longitudinal axis, while the arms swing opposite to the lower extremity motion to counterbalance the body angular momentum (Bruijn et al., 2008). These findings may explain why the arm swing increased with larger pelvic transverse rotation. In patients with PD who have both reduced trunk rotation and arm swing, training to increase pelvis transverse rotation may also improve their arm swing. The trunk moves in a sagittal plane during walking to counterbalance the lower extremity movement (Chung et al., 2010), while the arm swings to counterbalance the contralateral leg and the pelvic movement. Thus, the thoracic sagittal motion and arm swing both respond to the advancing movement of the lower limb. The regression analysis revealed that these two variables were closely associated. These preliminary findings need further investigations in aging and diseases in the future.

There are some discrepancies in the literatures due to different ethnic groups, subject age, walking conditions, testing equipment and techniques. With respect to studies on overground walking, Whittle & Levine (1999) studied twenty healthy young adult males using the vicon system and showed that the total range of motion in the pelvis was 7.72° ± 2.26° in the frontal plane, 2.79° ± 0.76° in the sagittal plane and 10.40° ± 3.22° in the transverse plane. Staszkiewicz et al. (2012) used Vicon 250 to measure pelvic ranges of motion of 30 men aged between 21 and 23 during natural gait at 5 km/h, and reported pelvic obliquity of 8.8° ± 2.18°, pelvic tilt of 2.2° ± 0.51°, pelvic rotation of 16.8° ± 4.43°. Crosbie, Vachalathiti & Smith (1997) studied 108 healthy adults whose ages ranged from 20 to 82 years using the video-based Expert-vision system and reported that the peak-to-peak ranges of motion in the pelvis during the free-speed gait cycle were 6.0° ± 2.5° in the coronal plane, 3.5° ± 1.5° in the sagittal plane, and 4.0° ± 2.5° in the transverse plane. The pelvic motions of older adults using APDM inertial sensor system were in general agreement with these literatures. As pelvic motions reduced with age, pelvic coronal and transverse ranges of motion of old adults were lower than previous reports regarding young adults. Pelvic sagittal range of motion in our study showed a slightly higher value. As to the studies on the treadmill, Stokes, Andersson & Forssberg (1989) used a SELSPOT system to assess the total range of motion in the thorax and pelvis across eight normal subjects at free speed and reported a pelvic coronal motion of 8.3° ± 2.3° , a sagittal motion of 3.9° ± 0.5°, a transverse motion of 7.9° ± 1.5°, a thoracic coronal motion of 4.7° ± 2.0°, a sagittal motion of 3.2° ± 0.9°, and a transverse motion of 4.6° ± 1.4°. Macpherson et al. (2016) quantified three-dimensional kinematics of the pelvic regions of nine male participants (29.2 ± 4.2 years) walking at self-selected speed (3.6–5.6 km/h) based on a single depth-sensing camera system, and reported pelvic frontal range of motion of 10.5° ± 1.1° and pelvic transversal range of motion of 6.3° ± 1.8°. In consistency with the finding (Staszkiewicz et al., 2012) that pelvic transverse range of motion on the ground was shown to be more than that in treadmill walking, our study revealed larger pelvic transverse plane motion compared to previous treadmill data. Besides, the thoracic motion on overground walking in our study was also larger than previous reports on the treadmill. These may be explained by the difference between treadmill and overground walking. Presumably, treadmill imposes constant walking speed that mechanically constrains fluctuations in stride lengths and stride times, which provides less opportunity to experience destabilizing perturbations and corresponding feedback necessary to ambulate overground. Moreover, optic flow on the treadmill is different from that during overground ambulation, which may also affect gait strategies since optic flow is used for locomotor control under varying conditions (Warren et al., 2001).

The limitations of this study should be considered. Linear regression models were fitted with the data (gait speed, angular ranges of motion and angular velocity), but other variables such as angular acceleration, jerk, joint moments and muscle forces may have a considerable effect as well. Thus, the regression models derived in this study can only be used to understand the relationships between the studied variables but not for the precise value prediction of each dependent variable.

## Conclusions

The present research studied age and gender difference of three-dimensional thoracic and pelvic movements and arm swing maximum velocity as well as the side preference of arm swing maximum velocity of healthy older adults during overground walking and provided insight into the relationships of these variables using the inertial sensor system, which will contribute to our better understanding of gait phenomena and have valuable implications in helping identify gait dysfunction and develop rehabilitation measures.

## Supplemental Information

10.7717/peerj.9329/supp-1 Supplemental Information 1 Dataset.

10.7717/peerj.9329/supp-2 Supplemental Information 2 Sensor placement. **Sternum**: on top of the sternum, with the top of the sensor located at the point separating the body of the sternum and the manubrium in reference to the thorax; **Lumbar**: center of the lower back (lumbar vertebrae 5 in reference to the pelvis); **Wrist**: On the wrist worn like a watch; **Foot**: Centered on top of the foot.

## Additional Information and Declarations

### Data Availability

The following information was supplied regarding data availability: The raw measurements are available as a Supplemental File.
