# Supplementary material for: Three-dimensional thoracic and pelvic kinematics and arm swing maximum velocity in older adults using inertial sensor system
Source: PeerJ. 2020 Jul 7;8:e9329. doi: 10.7717/peerj.9329 (PMC7350916; doi:10.7717/peerj.9329)
Supplement: Supplemental Information 1 [file peerj-08-9329-s001.doc]

**S1 Table. The data set of this manuscript.**

| **Subject** | **Gender** | **Age group**  **(years)** | **Gait Speed (m/s)** | **Pelvic Coronal Range of Motion (degrees)** | **Pelvic Sagittal Range of Motion (degrees)** | **Pelvic Transverse Range of Motion (degrees)** | **Thoracic Coronal Range of Motion (degrees)** | **Thoracic Sagittal Range of Motion (degrees)** | **Thoracic Transverse Range of Motion (degrees)** | **Left Arm/Maximum Velocity (degrees/s)** | **Right Arm/Maximum Velocity (degrees/s)** |
| --- | --- | --- | --- | --- | --- | --- | --- | --- | --- | --- | --- |
| 1 | Male | 80~89 | 0.520317 | 4.8058 | 4.13053 | 5.48772 | 6.16109 | 3.70741 | 10.3893 | 94.5123 | 106.306 |
| 2 | Male |  | 0.697717 | 3.64022 | 5.83353 | 8.94451 | 8.41462 | 7.34084 | 13.0119 | 242.926 | 182.215 |
| 3 | Male |  | 0.337826 | 2.98696 | 4.17329 | 6.96799 | 2.86709 | 4.35697 | 7.47882 | 86.9714 | 83.2998 |
| 4 | Male |  | 0.785535 | 2.6409 | 3.8527 | 6.30499 | 3.96527 | 3.9546 | 7.2605 | 101.88 | 117.56 |
| 5 | Male |  | 0.489681 | 2.78481 | 5.15246 | 4.82832 | 4.58142 | 4.65832 | 8.13326 | 93.7049 | 75.8084 |
| 6 | Male |  | 0.700167 | 3.0487 | 3.37104 | 8.13179 | 4.41588 | 4.49396 | 12.7939 | 164.919 | 93.1177 |
| 7 | Male |  | 0.77572 | 4.45191 | 3.51675 | 4.59631 | 4.47022 | 3.86559 | 10.6747 | 140.815 | 150.01 |
| 8 | Male |  | 0.641789 | 2.49559 | 4.1274 | 7.22817 | 7.15285 | 3.52535 | 10.2105 | 94.6603 | 82.5145 |
| 9 | Male |  | 1.17136 | 6.66669 | 2.65645 | 7.63237 | 4.18678 | 3.10986 | 9.68734 | 179.269 | 234.268 |
| 10 | Male |  | 0.762248 | 5.64983 | 2.59663 | 8.95822 | 6.23825 | 4.17853 | 14.2404 | 168.992 | 251.152 |
| 11 | Male |  | 0.678041 | 2.58681 | 4.36037 | 9.71252 | 4.34584 | 4.63673 | 12.203 | 95.7034 | 82.3756 |
| 12 | Male |  | 0.837831 | 4.06917 | 2.99499 | 11.5412 | 4.85943 | 3.7787 | 10.7829 | 199.989 | 192.769 |
| 13 | Male |  | 0.856226 | 5.64047 | 4.36992 | 6.55363 | 6.12395 | 4.39082 | 13.6181 | 125.192 | 156.889 |
| 14 | Male |  | 0.38577 | 3.57866 | 2.63411 | 5.68241 | 2.49953 | 3.05265 | 5.93414 | 54.4808 | 47.3964 |
| 15 | Male | 70~79 | 0.789484 | 4.54706 | 3.67067 | 4.27471 | 4.45916 | 4.11876 | 6.6678 | 121.861 | 114.591 |
| 16 | Male |  | 0.825605 | 6.35342 | 4.36869 | 8.6642 | 5.56241 | 6.81135 | 13.1998 | 285.299 | 223.851 |
| 17 | Male |  | 0.72505 | 3.10744 | 1.90647 | 5.26615 | 5.7373 | 2.64291 | 8.78961 | 123.67 | 80.3655 |
| 18 | Male |  | 1.15374 | 3.15991 | 2.36648 | 6.12888 | 3.25896 | 3.00769 | 8.2171 | 161.486 | 124.824 |
| 19 | Male |  | 0.943032 | 4.53413 | 2.80267 | 8.39974 | 6.90348 | 4.39756 | 9.68603 | 283.322 | 221.711 |
| 20 | Male |  | 1.14142 | 6.73828 | 4.31997 | 6.05415 | 7.95694 | 7.30253 | 8.50704 | 268.85 | 284.098 |
| 21 | Male |  | 1.25147 | 3.37658 | 5.25875 | 7.23237 | 3.81161 | 5.16469 | 11.0698 | 284.706 | 211.031 |
| 22 | Male |  | 0.695634 | 3.22929 | 3.95309 | 10.0739 | 3.18237 | 3.91115 | 12.1141 | 101.872 | 79.6833 |
| 23 | Male |  | 1.08162 | 4.05601 | 4.48793 | 4.67842 | 6.82321 | 4.32398 | 10.4093 | 159.978 | 225.532 |
| 24 | Male |  | 1.23172 | 5.04869 | 3.09419 | 8.52571 | 6.0284 | 3.53968 | 10.1642 | 114.857 | 165.297 |
| 25 | Male |  | 1.10869 | 3.90316 | 3.98689 | 4.39128 | 3.29479 | 3.63832 | 9.14116 | 115.779 | 164.38 |
| 26 | Male |  | 1.01998 | 6.08338 | 2.82649 | 9.38645 | 4.30211 | 3.93993 | 5.46757 | 229.836 | 226.8 |
| 27 | Male |  | 0.994339 | 4.86663 | 2.81255 | 5.91309 | 3.86542 | 2.88813 | 9.58244 | 86.2949 | 147.498 |
| 28 | Male |  | 0.975048 | 4.25173 | 3.05811 | 6.99326 | 5.4813 | 3.70417 | 6.13533 | 125.636 | 107.853 |
| 29 | Male |  | 1.14806 | 6.42206 | 4.84381 | 10.6541 | 7.70165 | 3.59316 | 5.38072 | 236.756 | 125.907 |
| 30 | Male |  | 1.10025 | 3.49464 | 4.95921 | 7.91994 | 3.32226 | 4.16035 | 9.48603 | 83.6222 | 99.5504 |
| 31 | Male | 60~69 | 0.807569 | 3.05134 | 3.51102 | 9.81086 | 5.93118 | 3.06079 | 9.54942 | 99.6849 | 87.2553 |
| 32 | Male |  | 1.23015 | 5.09431 | 3.54469 | 13.4495 | 8.72794 | 3.43068 | 7.71636 | 284.391 | 269.727 |
| 33 | Male |  | 1.02354 | 7.11217 | 5.2081 | 12.0467 | 6.38517 | 4.3558 | 13.0361 | 124.887 | 161.355 |
| 34 | Male |  | 1.05904 | 7.70754 | 4.28452 | 9.03172 | 4.67939 | 4.14543 | 9.62605 | 237.356 | 261.264 |
| 35 | Male |  | 1.30358 | 6.09048 | 3.44762 | 9.91329 | 7.8216 | 4.33808 | 6.00065 | 226.055 | 230.25 |
| 36 | Male |  | 1.11132 | 4.04406 | 3.67955 | 9.72081 | 7.05063 | 4.72794 | 12.0597 | 99.2861 | 96.8427 |
| 37 | Male |  | 1.09762 | 4.5332 | 3.30122 | 6.07631 | 6.78534 | 4.2721 | 12.6178 | 153.837 | 193.619 |
| 38 | Male |  | 1.19326 | 5.19823 | 2.96624 | 8.64732 | 6.62929 | 3.09172 | 7.06649 | 189.645 | 252.485 |
| 39 | Male |  | 0.979349 | 6.31333 | 4.11527 | 8.30925 | 3.9676 | 4.70745 | 8.05815 | 129.233 | 84.4972 |
| 40 | Male |  | 1.19526 | 5.52602 | 5.74001 | 7.96094 | 6.11887 | 6.63135 | 8.96897 | 185.068 | 240.414 |
| 41 | Male |  | 1.2117 | 3.03191 | 3.46861 | 7.3227 | 3.13974 | 3.8715 | 9.13182 | 180.801 | 225.643 |
| 42 | Male |  | 1.21961 | 7.48856 | 6.08284 | 4.9038 | 6.58112 | 4.59583 | 7.71554 | 164.135 | 162.181 |
| 43 | Male |  | 1.08017 | 4.03752 | 4.18048 | 7.70917 | 6.3083 | 3.67098 | 8.23873 | 112.966 | 137.401 |
| 44 | Male |  | 1.23841 | 5.45919 | 2.47448 | 10.0933 | 8.29638 | 3.08021 | 11.6901 | 194.098 | 81.6706 |
| 45 | Male |  | 1.02895 | 7.06419 | 3.53149 | 7.90756 | 3.31084 | 3.31101 | 9.13919 | 124.507 | 115.452 |
| 46 | Male |  | 1.12598 | 4.42446 | 4.47537 | 5.5364 | 3.83146 | 4.62064 | 9.38515 | 224.163 | 193.95 |
| 47 | Male |  | 1.25931 | 6.32012 | 3.58242 | 7.81352 | 8.1529 | 4.42771 | 9.47666 | 189.722 | 183.048 |
| 48 | Male |  | 0.927668 | 3.79509 | 4.25688 | 7.19161 | 2.74909 | 3.51839 | 11.5468 | 166.518 | 75.7581 |
| 49 | Male |  | 1.09892 | 3.20809 | 3.40827 | 6.95658 | 5.77679 | 3.24771 | 10.2623 | 148.541 | 155.285 |
| 50 | Male |  | 1.22933 | 8.75971 | 3.51279 | 13.5835 | 9.02515 | 5.72403 | 13.7398 | 289.807 | 330.489 |
| 51 | Female | 80~89 | 0.602474 | 2.75406 | 3.69329 | 8.16967 | 4.60168 | 3.60974 | 8.11595 | 109.312 | 90.8529 |
| 52 | Female |  | 0.716801 | 1.58732 | 4.57193 | 5.63045 | 3.08597 | 5.02847 | 7.47799 | 145.943 | 155.285 |
| 53 | Female |  | 0.695725 | 2.3955 | 2.73294 | 7.31127 | 4.72574 | 3.46636 | 10.2415 | 183.04 | 164.162 |
| 54 | Female |  | 0.746725 | 3.49475 | 3.18433 | 5.79136 | 3.1325 | 3.09618 | 8.22755 | 117.625 | 89.6808 |
| 55 | Female |  | 0.599537 | 5.3678 | 4.21206 | 11.954 | 5.31578 | 4.12378 | 11.3882 | 87.144 | 74.8283 |
| 56 | Female |  | 0.706735 | 3.6646 | 3.21791 | 5.54273 | 4.01103 | 3.20737 | 13.0218 | 96.1904 | 93.4741 |
| 57 | Female |  | 0.377011 | 4.01282 | 4.33352 | 11.8061 | 7.8211 | 4.18561 | 11.2126 | 198.665 | 182.039 |
| 58 | Female |  | 0.838491 | 6.46212 | 3.63986 | 4.08831 | 2.43821 | 4.34915 | 5.82404 | 94.617 | 92.4269 |
| 59 | Female |  | 0.490257 | 3.68724 | 3.92301 | 6.07249 | 3.37748 | 4.1192 | 8.82983 | 45.4132 | 42.7505 |
| 60 | Female |  | 0.897424 | 3.77317 | 4.13848 | 9.33014 | 6.12777 | 5.29634 | 8.70017 | 179.577 | 131.486 |
| 61 | Female |  | 0.880886 | 4.92146 | 3.53576 | 5.83996 | 4.8684 | 3.70379 | 6.22559 | 171.131 | 111.023 |
| 62 | Female |  | 0.446936 | 2.7704 | 5.00135 | 8.77644 | 4.238 | 6.13 | 10.1673 | 108.349 | 89.5647 |
| 63 | Female |  | 0.996216 | 5.45297 | 5.43656 | 9.41257 | 6.87551 | 4.97227 | 9.95141 | 146.762 | 135.317 |
| 64 | Female |  | 0.963994 | 5.44424 | 2.99576 | 8.99845 | 6.87128 | 3.59423 | 10.0643 | 153.45 | 166.555 |
| 65 | Female |  | 0.723599 | 2.69795 | 3.09258 | 12.3849 | 4.06828 | 4.30812 | 13.0677 | 171.072 | 192.345 |
| 66 | Female |  | 0.502695 | 3.48883 | 3.81879 | 9.43149 | 5.65363 | 3.77183 | 7.82125 | 88.593 | 60.8345 |
| 67 | Female |  | 1.02815 | 7.76524 | 3.15992 | 8.07002 | 7.05125 | 3.76934 | 14.9557 | 183.659 | 133.017 |
| 68 | Female |  | 0.887242 | 3.49898 | 3.63843 | 7.99466 | 6.31118 | 3.59085 | 8.76484 | 165.041 | 137.198 |
| 69 | Female |  | 0.687888 | 3.62423 | 4.36479 | 6.75201 | 2.40095 | 5.47255 | 10.8277 | 132.815 | 183.009 |
| 70 | Female |  | 0.487698 | 3.05724 | 3.5728 | 6.32586 | 3.38686 | 3.48683 | 6.76458 | 40.4572 | 62.5627 |
| 71 | Female | 70~79 | 0.98966 | 6.19434 | 4.46183 | 6.27779 | 7.91709 | 4.83708 | 11.8861 | 186.259 | 186.811 |
| 72 | Female |  | 1.09072 | 5.82568 | 4.55569 | 9.12448 | 4.22115 | 5.88675 | 9.12035 | 279.164 | 240.348 |
| 73 | Female |  | 0.806024 | 3.73005 | 3.80706 | 9.67175 | 4.37464 | 3.557 | 11.3358 | 112.42 | 92.264 |
| 74 | Female |  | 1.10184 | 4.93213 | 3.37156 | 5.17351 | 4.80473 | 3.32471 | 6.92668 | 136.164 | 117.059 |
| 75 | Female |  | 0.852338 | 5.62553 | 2.75029 | 12.1007 | 5.48882 | 4.02239 | 9.37408 | 164.744 | 136.388 |
| 76 | Female |  | 0.831572 | 2.66953 | 4.31803 | 6.25953 | 4.59959 | 3.32796 | 8.99181 | 169.172 | 182.955 |
| 77 | Female |  | 0.826216 | 3.45751 | 5.82776 | 4.53643 | 4.15403 | 5.4234 | 8.40873 | 112.935 | 107.398 |
| 78 | Female |  | 0.907477 | 7.03162 | 4.30148 | 7.61201 | 6.98599 | 5.52166 | 9.41693 | 170.414 | 151.954 |
| 79 | Female |  | 0.908292 | 5.74792 | 3.56686 | 7.56956 | 7.33452 | 2.72338 | 9.27146 | 145.512 | 149.154 |
| 80 | Female |  | 1.21429 | 6.54535 | 3.55385 | 11.8867 | 7.99568 | 5.01211 | 12.4758 | 201.83 | 203.558 |
| 81 | Female |  | 1.08612 | 8.60701 | 3.98258 | 6.17841 | 5.66315 | 5.32645 | 15.455 | 399.031 | 277.028 |
| 82 | Female |  | 1.10118 | 8.79505 | 3.50201 | 10.242 | 8.5361 | 3.36287 | 9.6814 | 238.724 | 239.02 |
| 83 | Female |  | 0.898952 | 4.3931 | 4.78323 | 9.1077 | 6.08345 | 4.53635 | 8.83896 | 131.616 | 118.782 |
| 84 | Female |  | 1.11646 | 3.08162 | 3.55423 | 9.3835 | 2.42967 | 4.87392 | 9.17249 | 211.745 | 123.981 |
| 85 | Female |  | 1.16249 | 7.03444 | 4.62254 | 5.64664 | 5.4718 | 6.6857 | 14.747 | 279.794 | 250.421 |
| 86 | Female |  | 0.820035 | 6.59606 | 4.56089 | 5.56276 | 4.64542 | 4.15974 | 8.68698 | 151.034 | 116.586 |
| 87 | Female |  | 0.694994 | 4.45527 | 4.04845 | 7.40959 | 4.9058 | 4.15893 | 9.68701 | 179.541 | 188.229 |
| 88 | Female |  | 1.22984 | 5.16722 | 5.37585 | 5.83053 | 4.97968 | 4.64581 | 16.8111 | 363.32 | 465.675 |
| 89 | Female | 60~69 | 1.12426 | 2.83318 | 4.44882 | 4.95884 | 4.74588 | 3.73372 | 9.44757 | 145.405 | 147.793 |
| 90 | Female |  | 1.10745 | 4.06804 | 3.10372 | 8.93972 | 5.12067 | 4.11578 | 6.18766 | 228.99 | 130.627 |
| 91 | Female |  | 1.23689 | 3.69325 | 5.22291 | 9.05959 | 7.96634 | 5.92837 | 9.01871 | 207.319 | 159.716 |
| 92 | Female |  | 1.09474 | 10.2532 | 4.67921 | 10.1255 | 8.47482 | 5.07664 | 6.97469 | 178.502 | 183.907 |
| 93 | Female |  | 1.07828 | 6.09838 | 3.61922 | 11.0839 | 7.92952 | 5.34429 | 5.45593 | 234.267 | 225.51 |
| 94 | Female |  | 0.999267 | 3.47091 | 4.09794 | 5.08358 | 2.91977 | 4.31823 | 8.76831 | 138.984 | 93.3837 |
| 95 | Female |  | 0.997452 | 7.6471 | 5.45472 | 7.03128 | 5.49428 | 5.19131 | 10.2196 | 279.692 | 213.997 |
| 96 | Female |  | 1.11059 | 1.91533 | 3.11799 | 6.0449 | 3.66094 | 2.99152 | 6.83475 | 105.215 | 148.72 |
| 97 | Female |  | 0.942736 | 3.75292 | 5.01685 | 6.30305 | 2.66958 | 4.60435 | 10.9392 | 132.898 | 131.996 |
| 98 | Female |  | 1.26911 | 5.93893 | 3.09171 | 9.33578 | 5.46135 | 3.08434 | 7.21891 | 199.139 | 186.801 |
| 99 | Female |  | 1.14052 | 5.97074 | 4.02307 | 9.37375 | 7.79744 | 3.92423 | 6.86572 | 215.695 | 231.617 |
| 100 | Female |  | 1.11351 | 6.01573 | 5.47272 | 11.0587 | 8.51063 | 5.58177 | 10.7797 | 265.359 | 241.617 |
| 101 | Female |  | 1.29876 | 4.70913 | 3.24928 | 7.89996 | 7.97323 | 5.7963 | 11.249 | 309.331 | 268.681 |
| 102 | Female |  | 1.29316 | 6.01478 | 4.35314 | 4.9212 | 4.91605 | 4.9635 | 7.5294 | 294.723 | 261.033 |
| 103 | Female |  | 1.02324 | 3.24336 | 4.6645 | 7.3612 | 3.02525 | 4.74665 | 13.6375 | 257.745 | 238.846 |
| 104 | Female |  | 1.17137 | 6.18241 | 4.38781 | 7.27919 | 4.90197 | 4.81745 | 8.16715 | 212.37 | 230.409 |
| 105 | Female |  | 1.14033 | 2.09251 | 6.06346 | 7.51208 | 3.86395 | 6.21176 | 10.031 | 144.517 | 199.727 |
| 106 | Female |  | 1.45252 | 6.31628 | 5.51473 | 8.52063 | 4.12803 | 5.52222 | 8.69906 | 304.651 | 215.672 |
| 107 | Female |  | 1.13508 | 4.78043 | 4.36542 | 4.42263 | 5.29689 | 4.42118 | 10.387 | 127.724 | 141.136 |
| 108 | Female |  | 1.1583 | 9.52721 | 6.54798 | 9.89492 | 8.89468 | 6.71829 | 12.5019 | 374.098 | 378.908 |
| 109 | Female |  | 1.21697 | 3.53028 | 4.10717 | 5.31461 | 4.09724 | 3.82714 | 8.14933 | 173.812 | 171.807 |
| 110 | Female |  | 1.24763 | 6.50113 | 4.29327 | 5.54531 | 4.63902 | 4.03872 | 10.711 | 225.482 | 220.716 |
| 111 | Female |  | 1.06348 | 4.81931 | 4.10615 | 6.81066 | 3.21209 | 3.8652 | 13.1849 | 165.985 | 148.934 |
| 112 | Female |  | 1.47457 | 5.77138 | 5.80842 | 7.45004 | 4.48731 | 5.87802 | 12.7691 | 156.322 | 148.375 |
| 113 | Female |  | 1.36795 | 9.6195 | 5.23723 | 18.6272 | 8.15574 | 5.22821 | 7.51472 | 473.038 | 574.007 |
