# Supplementary figures and images for: Three-dimensional thoracic and pelvic kinematics and arm swing maximum velocity in older adults using inertial sensor system
Source: PeerJ. 2020 Jul 7;8:e9329. doi: 10.7717/peerj.9329 (PMC7350916; doi:10.7717/peerj.9329)

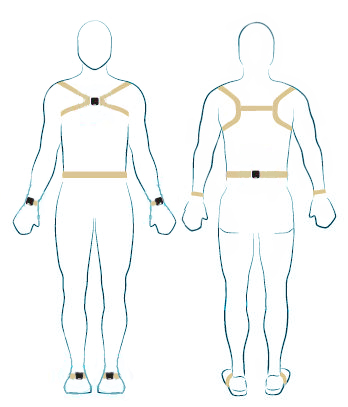

Supplement: Supplemental Information 2 — Sternum: on top of the sternum, with the top of the sensor located at the point separating the body of the sternum and the manubrium in reference to the thorax; Lumbar: center of the lower back (lumbar vertebrae 5 in reference to the pelvis); Wrist: On the wrist worn like a watch; Foot: Centered on top of the foot. [file peerj-08-9329-s002.jpg]
